# Increased Levels of Galectin-3 in Critical COVID-19

**DOI:** 10.3390/ijms242115833

**Published:** 2023-10-31

**Authors:** Ioanna Nikitopoulou, Alice G. Vassiliou, Nikolaos Athanasiou, Edison Jahaj, Karolina Akinosoglou, Ioanna Dimopoulou, Stylianos E. Orfanos, Vasiliki Dimakopoulou, Georgios Schinas, Argyrios Tzouvelekis, Vassilis Aidinis, Anastasia Kotanidou

**Affiliations:** 1 1st Department of Critical Care Medicine & Pulmonary Services, School of Medicine, National and Kapodistrian University of Athens, Evangelismos Hospital, 10676 Athens, Greece; joannaniki@gmail.com (I.N.); alvass75@gmail.com (A.G.V.); nikolaosathanasiou14@gmail.com (N.A.); edison.jahaj@gmail.com (E.J.); idimo@otenet.gr (I.D.); stylianosorfanosuoa@gmail.com (S.E.O.); 2 Division of Internal Medicine, University General Hospital of Patras, 26504 Patras, Greece; akin@upatras.gr (K.A.); dimakopoulou.vasilina@gmail.com (V.D.); georg.schinas@gmail.com (G.S.); 3 Department of Respiratory Medicine, University General Hospital of Patras, 26504 Patras, Greece; atzouvelekis@upatras.gr; 4 Institute of Fundamental Biomedical Research, Biomedical Sciences Research Center Alexander Fleming, 16672 Athens, Greece; aidinis@fleming.gr

**Keywords:** COVID-19, galectin-3, mortality, critically ill

## Abstract

Severe COVID-19 is related to hyperinflammation and multiple organ injury, including respiratory failure, thus requiring intensive care unit (ICU) admission. Galectin-3, a carbohydrate-binding protein exhibiting pleiotropic effects, has been previously recognized to participate in inflammation, the immune response to infections and fibrosis. The aim of this study was to evaluate the relationship between galectin-3 and the clinical severity of COVID-19, as well as assess the prognostic accuracy of galectin-3 for the probability of ICU mortality. The study included 235 COVID-19 patients with active disease, treated in two different Greek hospitals in total. Our results showed that median galectin-3 serum levels on admission were significantly increased in critical COVID-19 patients (7.2 ng/mL), as compared to the median levels of patients with less severe disease (2.9 ng/mL, *p* = 0.003). Galectin-3 levels of the non-survivors hospitalized in the ICU were significantly higher than those of the survivors (median 9.1 ng/mL versus 5.8 ng/mL, *p* = 0.001). The prognostic accuracy of galectin-3 for the probability of ICU mortality was studied with a receiver operating characteristic (ROC) curve and a multivariate analysis further demonstrated that galectin-3 concentration at hospital admission could be assumed as an independent risk factor associated with ICU mortality. Our results were validated with galectin-3 measurements in a second patient cohort from a different Greek university hospital. Our results, apart from strongly confirming and advancing previous knowledge with two patient cohorts, explore the possibility of predicting ICU mortality, which could provide useful information to clinicians. Therefore, galectin-3 seems to establish its involvement in the prognosis of hospitalized COVID-19 patients, suggesting that it could serve as a promising biomarker in critical COVID-19.

## 1. Introduction

Coronavirus disease 2019 (COVID-19) remains a health priority worldwide and despite the milder clinical course its severity can vary and still endanger patients upon hospitalization. Therefore, the determination of predictive biomarkers to classify a patient’s status could be useful for in-hospital management and high-risk patient optimal treatment. Patients with severe COVID-19 show imbalanced responses to infection, immunological cytokine alterations, lymphopenia, systemic inflammation including elevation of C-reactive protein, endothelial dysfunction, hyper-coagulation and tissue damage [1,2,3]. In this context, the evaluation of severe infection indicators is important to predict the clinically optimal prognosis. 

Galectin-3 belongs to a family of carbohydrate-binding proteins involved in many biological processes such as cell–cell and cell–matrix interactions, adhesion, proliferation, apoptosis, pre-mRNA splicing, immunity and inflammation [4]. Human galectin-3 is expressed by macrophages during phagocytosis and affects the differentiation and growth of various immune cells [5,6], playing a role in angiogenesis. Galectins can function as pattern recognition receptors and are found in various immune cells and tissues, where they play potential and significant roles in human infections caused by different types of pathogens [7]. Galectin-3 is also an important mediator in the exacerbation of autoimmune, inflammatory and malignant diseases [8,9]. Notably, galectin-3 is considered a pro-fibrotic molecule, elevated in the plasma and broncho-alveolar lavage fluid (BALF) of patients with idiopathic pulmonary fibrosis (IPF) [10]. This profibrotic function is related to the ability of galectin-3 to promote signaling through integrins and growth factor receptors, such as transforming growth factor-β, vascular endothelial growth factor and platelet-derived growth factor (PDGF) receptors [10,11].

In order to establish the prognostic value of galectin-3 at the clinical level in COVID-19 and to analyze the possibility of predicting the clinical course of patients, galectin-3 concentrations on admission in COVID-19 cases of various severity were determined, in comparison to other approved biomarkers and appropriate laboratory tests. 

## 2. Results

### 2.1. Increased Serum Galectin-3 Protein Levels in Severe COVID-19 Patients

**Figure 1:**
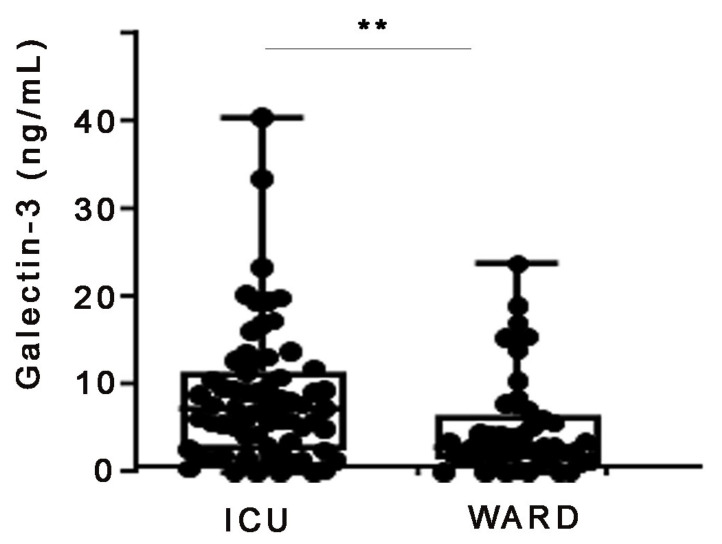
Increased serum admission galectin-3 levels in COVID-19 patients hospitalized in the intensive care unit (ICU) of Evangelismos hospital. Protein levels were measured with a commercial ELISA kit in the sera of ICU (*n* = 67) and WARD (*n* = 49) COVID-19 patients. ** denotes *p* < 0.01.

To examine whether systemic levels of galectin- 3 are increased upon COVID-19, galectin-3 was quantified with an ELISA kit (R&D Systems) in the serum of COVID-19 patients hospitalized at the Evangelismos University Hospital (Table 1 and Figure S1). The cohort consisted of ward (*n* = 49), as well as intensive care unit (ICU) patients (*n* = 67). COVID-19-targeted treatment was based on WHO recommendations at that time (Table 1). In comparison with ward patients, ICU patients had increased levels of C-reactive protein (CRP), lactate dehydrogenase (LDH) and ferritin, which are considered disease severity markers. Increased galectin-3 serum concentrations on admission were discovered in ICU patients as compared with ward patients (Figure 1), suggesting a possible association of galectin-3 with disease severity. 

### 2.2. Increased Levels of Galectin-3 Correlate with Poor ICU Prognosis

In order to investigate the prognostic utility of galectin-3 in critical COVID-19, we then focused on the patient group hospitalized in the ICU, which was further divided in survivors and non-survivors (Table 2). 

**Figure 2:**
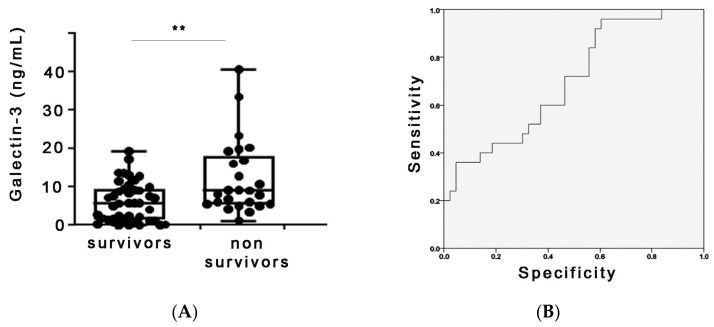
Galectin-3 serum admission levels negatively correlate with survival in COVID-19 patients hospitalized in the ICU of Evangelismos hospital. (**A**) Protein levels were measured, with a commercial ELISA kit. ** denotes *p* < 0.01 (**B**) A receiver operating characteristic (ROC) curve was generated, showing the prognostic accuracy of galectin-3 for the probability of ICU mortality.

Galectin-3 serum admission levels were found to negatively correlate with the survival of COVID-19 patients in the ICU. More specifically, the median galectin-3 levels of the non-survivors hospitalized in the ICU were significantly higher than those of the survivors (Figure 2A). For survivors, the median was 5.8 [interquartile range (IQR) 1.5–9.6] ng/mL, whereas median galectin-3 levels for the non-survivors were 9.1 (5.6–18.1, *p* = 0.001) ng/mL. Correlation analysis between galectin-3 and COVID-19 biomarkers showed a significant correlation with the serum levels of D-dimers, LDH, ferritin and CRP (Figure S2). A receiver operating characteristic (ROC) curve was then generated to examine the possible prognostic accuracy of galectin-3 in our cohort (Figure 2B). The area under the ROC curve (AUC) and 95% CI for detecting the main outcome, i.e., the ICU mortality, were estimated. A cut-off value of 7.75 ng/mL for galectin-3 was associated with a sensitivity of 61% and a specificity of 64.5% (AUC: 0.697; 95% CI: 0.569–0.823) for predicting ICU mortality. The univariate Cox regression survival analysis model for galectin-3 showed that galectin-3 values correlated with an increased risk of ICU mortality [OR: 1.044 (95% C.I. = 1.083–1.007), *p* = 0.018] (Table 3).

In the multivariate analysis adjusted for age, the APACHE II score and LDH demonstrated that galectin-3 concentration could be assumed as an independent risk factor related to ICU mortality [1.069 (1.128–1.013), *p* = 0.016] (Table 3). 

### 2.3. Validation of the Role of Galectin-3 in COVID-19 Severity

**Figure 3:**
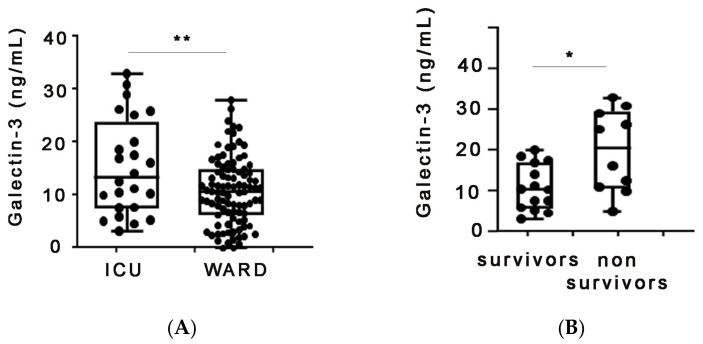
Verification of our results in a second, independent patient cohort. (**A**) Increased serum galectin-3 levels in COVID-19 patients hospitalized in the ICU of Patras University hospital. (**B**) Galectin-3 serum protein levels negatively correlate with survival in COVID19 patients hospitalized in the ICU of Patras University Hospital. * denotes *p* < 0.05 and ** denotes *p* < 0.01.

In order to validate our results, a multicenter study would be needed. For this reason, we measured galectin-3 in a second patient cohort from a university hospital in a different Greek area. More specifically, galectin-3 was quantified in the serum of COVID-19 patients hospitalized at the Patras University Hospital. The cohort consisted of ward (*n* = 95) as well as ICU patients (*n* = 24) (Table 4). Measurement results from this cohort were similar, as serum galectin-3 was also found to be increased in ICU patients compared to ward patients (Figure 3A). Notably, galectin-3 serum levels in the non-survivors hospitalized in the ICU of the Patras cohort were again significantly higher than those of survivors (Figure 3B).

## 3. Discussion

In this study, we demonstrated the association of serum galectin-3 concentration with COVID-19 severity. A significant increase in galectin-3 levels and admission levels in critically ill COVID-19 patients compared to patients with moderate disease was shown. Furthermore, our study focused on ICU mortality, showing that COVID-19 ICU non-survivors from the two different hospitals had significantly higher galectin-3 levels compared to survivors. Our data point out that galectin-3 is an independent risk factor related to ICU mortality, highlighting its importance in disease severity as well as in outcome. Although a link between galectin-3 and the clinical course of COVID-19 has been reported in a few studies [12,13], to the best of our knowledge, this is the first report verifying this association in patient cohorts from two different centers, therefore strengthening the notion that galectin-3 is an important mediator in COVID-19 progression. Previous studies addressing the role of galectin-3 in COVID-19 have focused on diagnosing COVID-19 pneumonia or the need for mechanical ventilation [14,15,16]. More specifically, Kusnierz-Cabala et al. showed that galectin-3 had moderate diagnostic accuracy for COVID-19 pneumonia and high diagnostic accuracy for the need for ICU treatment [16]. The same study revealed positive correlations between galectin-3 and several commonly studied inflammatory markers, such as CRP, IL-6 and ferritin. In addition, results from a study by Ozcan et al. indicated that galectin-3, IL-6 and CRP levels on hospital admission are related to the need for transfer to the ICU and that galectin-3 alone is related to the need for advanced ventilatory support [14]. Galectin-3 was also studied in the context of thrombogenicity and was found to significantly correlate with von Willebrand Factor (vWF) and mean platelet volume (MPV) in a COVID-positive group as compared to a COVID-negative group, thus indicating platelet activation [17]. Our results confirm previous studies on galectin-3, using not only larger patient numbers, but two cohorts from different catchment areas. The possibility to predict an adverse clinical course and/or death was central in our study since it could provide useful information to clinicians.

Galectins are implicated in infectious diseases, as they have been previously shown to participate in immunological responses to infections through their function as a recognition and effector factor in innate immunity [18]. They are widely expressed in mammalian tissues, including cells of the immune system (dendritic cells, macrophages, mast cells, natural killer cells and activated B and T cells), and are known to bind glycans on the surface of bacteria, viruses, and fungi [19]. Galectin-3, in particular, can respond to damaged phagosomes during *Mycobacterium tuberculosis* infection [20] and can also function as a macrophage receptor for *Candida albicans* [21]. Concerning the role of galectin-3 in viral infections, it can participate in interactions taking place between virus and host during viral entry, viral replication, or immune response modulation [7]. Hepatitis C virus infection increases serum galectin-3 levels, which in turn can promote inflammation and fibrosis progression [22]. Galectin-3 is also suggested to play a role in the early stages of human immunodeficiency virus (HIV) infection by enhancing viral entry [23]. Other studies involving human viral infections such as the herpes simplex virus and the varicella-zoster virus suggest a role for galectin-3 [24,25], with a specific focus on the regulation of adaptive immunity [26]. More specifically, in pneumonia diagnosis, it has already been suggested that galectin-3 can trigger influenza-induced pulmonary inflammation through inflammasome activation [27]. In this context, our results on increased levels of serum galectin-3 in response to SARS-CoV2 infection are in agreement with previous knowledge. Concerning the serum levels of the protein measured in our cohort, they appeared higher than those reported previously in healthy donors (mean 3.8 ng/mL) [28]. 

In severe COVID-19, pulmonary fibrosis is considered a disease risk and a possible complication [29,30]. Fibrosis is also present in fatal case autopsies of COVID-19 [31]. Serum galectin-3 has been proposed as a key player in chronic inflammatory states associated with fibrosis [25,32]. More specifically, the role of galectin-3 as a mediator of pulmonary fibrosis has drawn attention since the inhibition of galectin-3 was found to decrease bleomycin-induced fibrosis and lung inflammation in mice [33]. Notably, galectin-3 levels are also elevated in BALF and serum of IPF patients [10,34], with inhaled administration of a galectin-3 inhibitor showing promising results in IPF progression [35]. COVID-19 and pulmonary fibrosis share similarities, and therapeutic strategy could be commonly applied, alone or in combination with approved antifibrotic or antiviral therapy. Previous results have shown that galectin-3 is upregulated in proliferative T lymphocytes associated with severe COVID-19 and that a subset of pro-fibrogenic macrophages also expresses biomarkers strongly related to galectin-3 [36]. Overall, given the role of galectin-3 in immunomodulation and inflammatory response, this protein may represent a prominent pharmacological target for lung fibrosis in COVID-19 patients [25]. Of note, a very recent phase Ib/IIa randomized controlled platform trial (NCT04473053) in hospitalized patients with confirmed COVID-19 pneumonitis showed that a potent inhaled thiodigalactoside galectin-3 inhibitor (GB0139) was well-tolerated and reached clinically relevant plasma concentrations with target engagement [37]. These data apart from demonstrating safety and tolerability of the inhaled inhibitor, will hopefully lead to large clinical trials. 

Although the relationship between galectin-3 and COVID-19 has been studied before, in this study we mainly aimed to explore its correlation with worse outcomes and increased mortality in the ICU. Sensitivity and specificity percentages are not very high and could be considered a limitation; however, after the multivariate analysis, the only parameter remaining significant was galectin-3. Another limitation of our study is that it did not focus on different SARS-CoV-2 variants or vaccination strategies. Nevertheless, it involved a two-center patient cohort with a large patient number. Even though the mechanism underlying our findings is not clear, these data were verified in two Greek University hospitals, thus supporting published work and linking galectin-3 to disease severity. Forthcoming studies focusing on the link between galectin-3 and long COVID-19 will further establish its role in the pathophysiology of this disease. 

Conclusively, our results point out that galectin-3 is related to disease severity, and its high concentrations are linked to worse outcomes and increased mortality in the ICU. Altogether, these data suggest that galectin-3 could be considered a possible therapeutic target in COVID-19.

## 4. Materials and Methods

### 4.1. Study Design and Data Collection

The study was approved by the Evangelismos Hospital Research Ethics Committee (170/24-4-2020) and all procedures carried out on patients were in compliance with the Helsinki Declaration. Written informed consent was obtained from all patients’ next-of-kin. All consecutive patients hospitalized at Evangelismos General Hospital with a diagnosis of COVID-19 (confirmed by real-time reverse transcription PCR in nasopharyngeal swabs) between March and May 2020 were included in this prospective, observational study. Following study enrolment, demographic characteristics, comorbidities, symptoms and laboratory findings were recorded. 15 patients had mild disease, 25 patients had moderate disease, 8 patients had severe disease and 67 patients had critical disease. Acute Physiology and Chronic Health Evaluation (APACHE II) and Sequential Organ Failure Assessment (SOFA) scores were calculated on ICU admission. Laboratory tests were performed within 24 h of admission.

The study also included a cohort of COVID-19 patients admitted to University Hospital of Patras from April to December 2020. The study was approved by the University Hospital of Patras Research Ethics Committee (#216/08-05-2020). All consecutive patients hospitalized with a diagnosis of COVID-19 were included in the study. Following enrolment, demographic characteristics, comorbidities, symptoms and laboratory findings were recorded.

Galectin-3 protein levels were quantified on admission in serum samples with an ELISA kit according to the manufacturer’s instructions (R&D Systems Inc., Minneapolis, MN, USA). 

### 4.2. Statistical Analysis

Data are presented as mean ± standard deviation (SD) for normally distributed variables or as median with inter-quartile range (Q1–Q3) for skewed data. The two-group comparisons were assessed by the *t*-test or the non-parametric Mann–Whitney test, as appropriate. Associations between qualitative variables were examined by the chi-square test and correlations were evaluated by Spearman’s correlation test. Receiver operating characteristic (ROC) curves used ICU mortality as the classification variable. Univariate and multivariate Cox regression analyses were performed to identify potential risk factors for ICU mortality. Analyses were performed with the GraphPad Prism software version 8.0 (GraphPad Software, San Diego, CA, USA) and the statistical Package for the Social Sciences 19.0 (SPSS Inc., Chicago, IL, USA). All *p*-values are two-sided; *p* < 0.05 was considered significant. 

## 5. Conclusions

Increased galectin-3 in serum was found to be associated with critical illness severity in patients with COVID-19, and the highest concentrations were further associated with the most severe disease progression and ICU mortality. Our findings were verified in two Greek university hospitals from different areas, thus suggesting a major role of galectin-3 in the pathogenesis of the disease. Taking into consideration the involvement of galectin-3 in the hyperinflammation and fibrosis stages of the COVID-19 clinical course, new therapeutic approaches useful in severe cases could be suggested.

## Figures and Tables

**Table 1:** Clinical characteristics and laboratory data of patients hospitalized in Evangelismos that were included in the study.

Patient Clinical Characteristics	ICU	Ward
Number of patients (*n*)	67	49
Sex		
Male	51 (76.1%)	33 (67.3%)
Female	16 (23.9%)	16 (32.7%)
Age (years, mean ± SD)	64.3 ± 10.5	56.6 ± 16
Comorbidities *n* (%)		
Hypertension	23 (34.3%)	9 (18.4%)
Diabetes	7 (10.4%)	4 (8.1%)
Hyperlipidaemia	16 (23.8%)	9 (18.4%)
COPD	3 (4.4%)	2 (4%)
Chronic kidney disease	0	0
Coronary artery disease	7 (10.4%)	8 (16.3%)
COVID 19 treatment		
Azithromycin/chloroquine/		
lopinavir/ritonavir	11	
Azithromycin/chloroquine	7	6
Lopinavir/ritonavir/		
chloroquine	2	
Chloroquine	3	
Azithromycin	8	29
Plasma	1	
Mechanical Ventilation (days), (median, IQR)	11 (4–30)	N/A
Hospital mortality (%)	31.3	8.2 *
Hospital length of stay (days), (median, IQR)	18 (12–31)	9 (7–12)
Days sick before admission (median, IQR)	7 (5–8)	6 (5–9)
Diffuse lung infiltrates in X-ray	62 (92.5%)	30 (61.2%)
Pneumonia	67 (100%)	34 (69.4%)
Glucose (mg/dL) (median, IQR)	141 (108–204)	101 (82–127) *
Creatinine (mg/dL)	1.02 ± 0.32	0.9 ± 0.33
CRP (mg/dL), (median, IQR)	13.3 (5.3–21)	6.4 (2–15) *
Na^+^ (mEq/L)	140 ± 7	135 ± 4
Total bilirubin (mg/dL)	0.6 (0.4–0.88)	0.4 (0.3–0.57)
White blood cell count (per μL)	10,300 (8200–13,200)	6900 (4600–9800)
Neutrophils (%)	82.5 ± 11.4	69.2 ± 13.2
Lymphocytes (%)	12.5 ± 6.34	24.03 ± 10.89
D-dimer (pg/mL)	1.71 ± 0.29	1.14 ± 0.23
LDH (U/L), (median, IQR)	422 (334–590)	263 (209–366) *
Fibrinogen (mg/dL	638 ± 168	521 ± 174
Ferritin (pg/mL), (median, IQR)	621 (198–1124)	264 (108–513) *

Data are presented as individual numbers, *n* (%), mean± SD or median (IQR). Comparisons between groups were performed using the Mann–Whitney test. COPD: chronic obstructive pulmonary disease; CRP: C-reactive protein; ICU: Intensive Care Unit; LDH: lactate dehydrogenase; N/A: not applicable. * denotes *p* < 0.05. ICU group, critical disease; Ward group, mild-severe patients.

**Table 2:** Clinical characteristics and laboratory data of patients hospitalized in the ICU of Evangelismos hospital.

Patient Clinical Characteristics	Survivors	Non-Survivors
Number of patients (*n*)	42	25
Sex		
Male	30 (71.4%)	21 (84%)
Female	12 (28.6%)	4 (16%)
Age (years, mean ± SD)	61.5 ± 10.1	68.3 ± 9.3
SOFA (median, IQR)	6 (4–8)	7 (4–9)
APACHE II (median, IQR)	15 (11–17)	17 (14–18) *
Mechanical Ventilation (days) (median, IQR)	11 (4–30)	24 (12–31)
D-dimer (pg/mL)	1.54 ± 2	2 ± 2.2
LDH (U/L)	414 (322–498)	532 (375–726) *
CRP (mg/dL)	11.3 (5–18.4)	15 (5.3–27)
Ferritin (pg/mL)	576 (117–1850)	744 (486–2740)

Data are presented as individual numbers, *n* (%), mean± SD or median (IQR). Comparisons between groups were performed using the Mann–Whitney test. APACHE: acute physiology and chronic health evaluation; SOFA: sequential organ failure assessment; CRP: C-reactive protein; LDH: lactate dehydrogenase. Laboratory tests were performed within 24 h of admission. * denotes *p* < 0.05.

**Table 3:** Univariate and multivariate Cox regression analysis.

Variable	Univariate Model		Multivariate Model	
	OR (95%CI)	*p*-Value	OR (95%CI)	*p*-Value
Galectin-3	1.044 (1.007–1.083)	0.018	1.069 (1.013–1.128)	0.016
Age	1.018 (0.980–1.057)	0.359	1.016 (0.974–1.060)	0.465
LDH	1.001 (1.000–1.002)	0.053	1.001 (1.000–1.002)	0.065
APACHE	1.008 (0.915–1.110)	0.878	1.014 (0.904–1.138)	0.810

A univariate Cox regression model was fitted to examine the relationship of galectin-3 admission levels with ICU mortality. Multivariate regression analysis revealed that galectin-3 concentration could be assumed as an independent indicators of ICU mortality. C.I: confidence interval; O.R.: odds ratio; APACHE: acute physiology and chronic health evaluation; LDH: lactate dehydrogenase.

**Table 4:** Clinical characteristics and laboratory data of patients hospitalized in Patras General hospital that were included in the study.

Patient Clinical Characteristics	ICU	Ward
Number of patients (*n*)	24	95
Sex		
Male	20 (83.3%)	49 (51.6%)
Female	4 (16.7%)	46 (48.4%)
Mechanical Ventilation (days) (median, IQR)	15 (6–23)	N/A
Hospital mortality (%)	41.6	4.2 *
Comorbidities n (%)		
Hypertension	5 (20.8%)	44 (46.3%)
Diabetes	0 (0%)	18 (18.9%)
Hyperlipidaemia	3 (12.5%)	38 (40%)
Coronary artery disease	3 (12.5%)	5 (5.2%)
COPD	2 (8.3%)	4 (4.2%)
Age (years, mean ± SD)	62.3 ± 15.5	59 ± 15
Hospital length of stay (days), (median, IQR)	20 (12–25)	9 (6–12)
Days sick before admission (median, IQR)	7 (5–9)	6 (3–7)
Diffuse lung infiltrates in X-ray	23 (95.8%)	74 (77.9%)
Glucose (mg/dL) (median, IQR)	127 (102–165)	115 (101–138)
Creatinine (mg/dL)	1.14 ± 1.28	0.95 ± 0.41
CRP (mg/dL) (median, IQR)	12.3 (4.5–16)	4 (1.5–8.2) *
Na^+^ (mEq/L)	139 ± 5.8	136 ± 3.3
Total bilirubin (mg/dL) (median, IQR)	0.67 (0.46–0.94)	0.5 (0.39–0.66)
White blood cell count (per μL) (median, IQR)	7000 (4000–9700)	5400 (4300–7000)
LDH (U/L) (median, IQR)	412 (280–522)	289 (246–355) *
D-dimer (pg/mL)	1.67 ± 1.96	1.12 ± 1.6 *
Fibrinogen (mg/dL)	799 ± 120	530 ± 133
Ferritin (pg/mL) (median, IQR)	855 (411–1720)	351 (190–745) *

Data are presented as individual numbers, *n* (%), mean± SD or median (IQR). Comparisons between groups were performed using the Mann–Whitney test. COPD: chronic obstructive pulmonary disease; CRP: C-reactive protein; LDH: lactate dehydrogenase; ICU: Intensive Care Unit; N/A: not applicable. * denotes *p* value < 0.05.

## Data Availability

Data are available upon reasonable request.

## Supplementary Materials

The following supporting information can be downloaded at: https://www.mdpi.com/article/10.3390/ijms242115833/s1.
