# Supplementary figures and images for: Increased Levels of Galectin-3 in Critical COVID-19
Source: Int J Mol Sci. 2023 Oct 31;24(21):15833. doi: 10.3390/ijms242115833 (PMC10650562; doi:10.3390/ijms242115833)

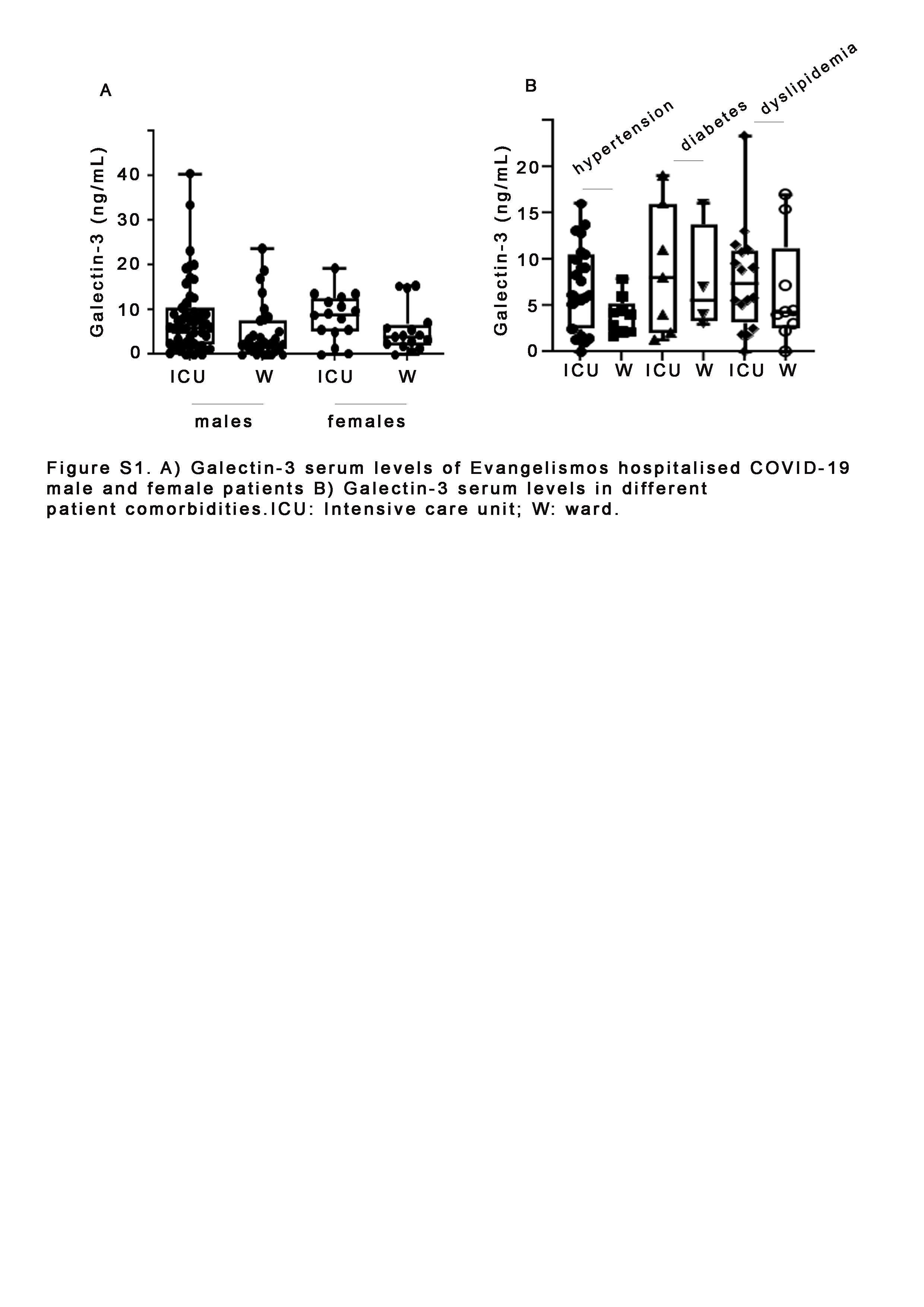

Supplement: Supplementary file 1 [file ijms-24-15833-s001.zip › Fig S1.tif]

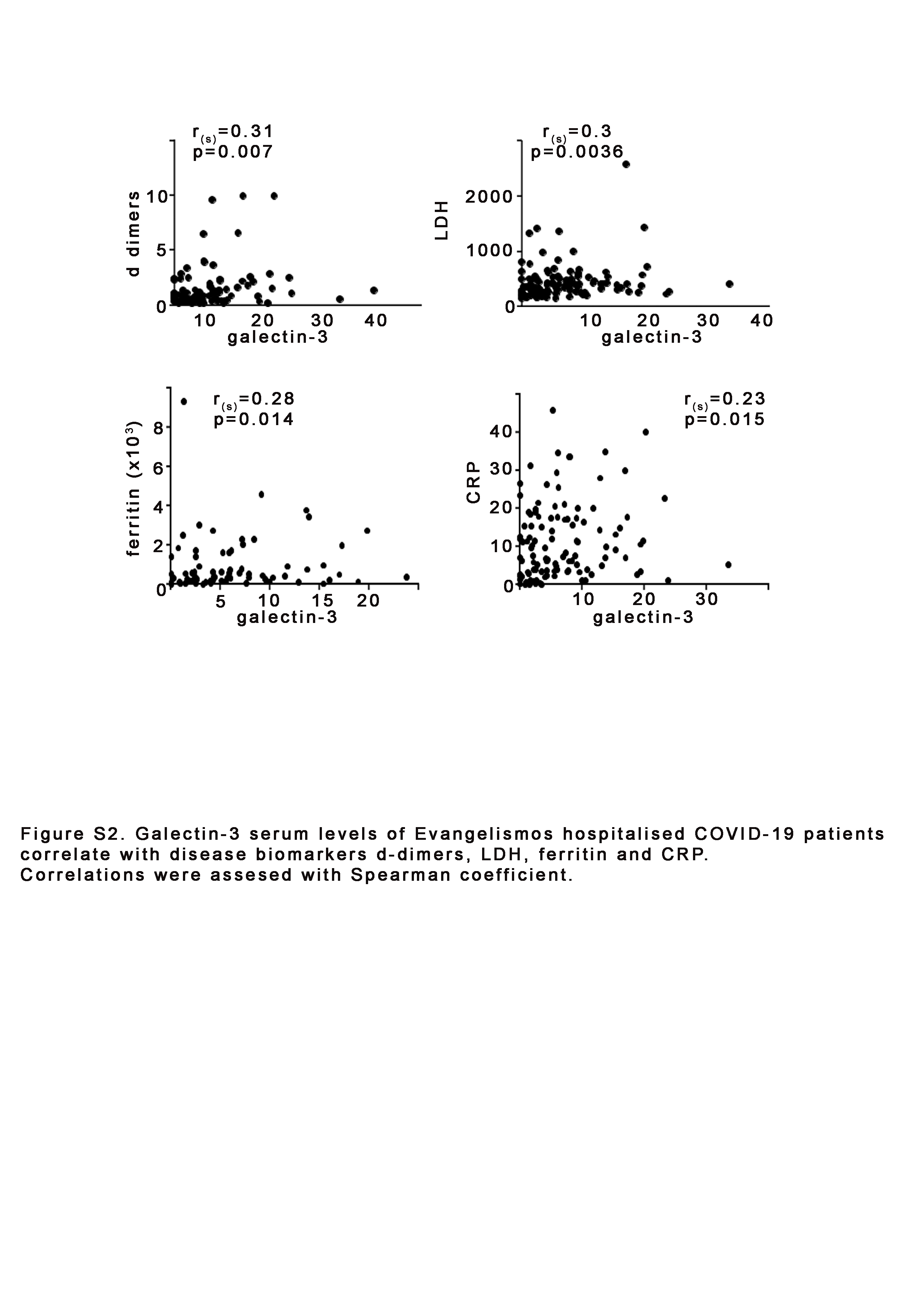

Supplement: Supplementary file 1 [file ijms-24-15833-s001.zip › Fig S2.tif]
